# One Century of Study: What We Learned about *Paracoccidioides* and How This Pathogen Contributed to Advances in Antifungal Therapy

**DOI:** 10.3390/jof7020106

**Published:** 2021-02-02

**Authors:** Erika Seki Kioshima, Patrícia de Souza Bonfim de Mendonça, Marcus de Melo Teixeira, Isis Regina Grenier Capoci, André Amaral, Franciele Abigail Vilugron Rodrigues-Vendramini, Bruna Lauton Simões, Ana Karina Rodrigues Abadio, Larissa Fernandes Matos, Maria Sueli Soares Felipe

**Affiliations:** 1 Program in Biosciences and Pathophysiology, Department of Clinical Analysis and Biomedicine, State University of Maringa (UEM), Maringa, Parana 87020-900, Brazil; patbonfim.09@gmail.com (P.d.S.B.d.M.); isiscapoci@gmail.com (I.R.G.C.); francieleavr@gmail.com (F.A.V.R.-V.); blautons@gmail.com (B.L.S.); 2 Faculty of Medicine, University of Brasília (UnB), Brasilia, Distrito Federal 70910-900, Brazil; marcus.teixeira@gmail.com; 3 Institute of Tropical Pathology and Public Health, Federal University of Goiás, Goiânia 74690-900, Brazil; amaral.nanobio@gmail.com; 4 Faculty of Agricultural Social Sciences, Mato Grosso State University, Nova Mutum, Mato Grosso 78450-000, Brazil; anakarina.abadio@gmail.com; 5 Faculty of Ceilandia, University of Brasília (UnB), Brasília, Distrito Federal 72220-275, Brazil; lfernandesmatos@gmail.com; 6 Program in Microbial Biology, Institute of Biological Sciences, University of Brasília, Brasília 70910-900, Brazil; 7 Program of Genomic Sciences and Biotechnology, Catholic University of Brasilia, Brasília 70790-160, Brazil; msueli@ucb.br

**Keywords:** genomic, new species discovery, new targets, antifungal

## Abstract

Paracoccidioidomycosis (PCM) is a notable fungal infection restricted to Latin America. Since the first description of the disease by Lutz up to the present day, Brazilian researchers have contributed to the understanding of the life cycle of this pathogen and provided the possibility of new targets for antifungal therapy based on the structural and functional genomics of *Paracoccidioides*. In this context, *in silico* approaches have selected molecules that act on specific targets, such as the thioredoxin system, with promising antifungal activity against *Paracoccidioides*. Some of these are already in advanced development stages. In addition, the application of nanostructured systems has addressed issues related to the high toxicity of conventional PCM therapy. Thus, the contribution of molecular biology and biotechnology to the advances achieved is unquestionable. However, it is still necessary to transcend the boundaries of synthetic chemistry, pharmaco-technics, and pharmacodynamics, aiming to turn promising molecules into newly available drugs for the treatment of fungal diseases.

## 1. From the First Description of the Disease to Functional Genomics—112 Years of Gathered Knowledge. A Brazilian Middle West Perspective

First described in 1908 by Adolpho Lutz as “A Pseudococcidic Mycosis Localized in the Mouth and Observed in Brazil” [1], paracoccidioidomycosis is the most prevalent systemic mycosis in South America. This fungal disease was named Brazilian blastomycosis, South American blastomycosis, paracoccidioidal granuloma, Lutz or Lutz-Splendore-Almeida disease but only in 1971 was it officially named paracoccidioidomycosis after a meeting of medical mycologists in Medellin, Colombia [2]. PCM was confused with coccidioidomycosis for about two decades. However, after a precise microbiological characterization of the dimorphic and multi-budding nature of the fungus, the disease was ranked as a new mycosis by identifying its etiological agent as *Paracoccidioides brasiliensis* [3]. Historically, the disease has been extensively investigated in the Brazilian Southeast, Colombia and Venezuela regarding the etiological agent, epidemiological characteristics, disease variation, immunological aspects, cell wall components, diagnostics and treatment, as well as the biological aspects of this saprophytic fungus.

PCM primarily affects the lungs upon the inhalation of airborne infectious propagules named conidia. These cells produced during the filamentous phase of the fungus are dislodged and aerosolized after soil perturbation. Most natural infections are asymptomatic and characterized by a self-resolved disease in humans and other mammalian hosts [4]. Inside the host lungs, the fungus switches its morphology to multi-budding yeast cells, triggering the infection and leading to an acute/subacute disease (also known as the juvenile form) predominantly reported in infants/adolescents, or into a chronic-to-disseminated polymorphic disease. A detailed review can be found at Shikanai-Yasuda et al., 2017 [5].

Past relevant studies carried out by Willian Barbosa, comparing clinical PCM in different parts of Brazil, suggested that lymphatic-abdominal forms of *Paracoccidioides* were predominant among patients in Midwestern, especially in Goiás state, compared to Southeastern states [6,7]. The Brazilian Midwest is composed of the Federal District and Goiás, Mato Grosso and Mato Grosso do Sul states, harboring two predominant ecosystems (Brazilian Savanna and Pantanal) and is a key ecological area of transition to the Amazon. These observations drove several research groups to investigate local clinical and microbiological determinants of PCM in Brazil. It is worth noting that in the last three decades, there has been massive deforestation of the Brazilian savanna and Amazon landscapes in Brazil and surrounding countries due to agricultural practices and pasture [8,9]. Consequently, PCM cases increased in those areas, redrawing the epidemiological map of this fungal disease. A discussion of this topic can be found in Martinez, 2017 [8]. Notably, those patients from Goiás, Mato Grosso and Rondônia (Midwestern Brazil) had apparent clinical symptomatology of PCM and microbiological features of *Paracoccidioides*, but a high percentage of negative serological tests was observed by utilizing the well-characterized Pb339 antigen obtained from a strain isolated in São Paulo (Southeast) [10,11,12]. An antigenic preparation using a local strain, named 510B, was proposed and was crucial to improving antibody detection assays in Midwestern Brazil [12].

Although studies had already indicated a high degree of genetic divergence among isolates of *P. brasiliensis*, at that moment there was no geographical correlation with these findings [13,14,15]. By Random Amplification of Polymorphic DNA (RAPD) markers, Molinari-Madlum et al. [13] were able to identify two groups with different virulence degree and a high of genetic diversity. Despite the study focusing on the analysis of genetic markers related to virulence, the Pb01 isolate already presented a different pattern to well-known *P. brasiliensis* isolates, such as Pb18 [13]. The RAPD studies continued to show high genetic variability among clinical isolates, including atypical forms [14]. Next, Hahn et al. [15] reported the same observations comparing the RAPD profiles of Mato Grosso and other areas of the disease range in Brazil. The genetic variability was characterized, but it was not possible to establish a geographical differentiation pattern [15]. Thus, despite the suspicions of clinicians, who had already observed in practice that *P. brasiliensis* isolates from the Midwestern were different, confirmation was only possible later with advances in fungal genotyping methods.

At the beginning of the 2000s, fungal systematics and taxonomy dramatically changed when the phylogenetic species concept (PSC) was implemented, which relied on the phylogenetic concordance of several loci in a given population [16,17]. PSC was used to delineate phylogenetic species with *Histoplasma* sp., *Coccidioides* sp., and at least three phylogenetic species diagnosed with *P. brasiliensis*: S1—widely distributed in Latin America; PS2—less abundant and prevalent in southeastern Brazil and Venezuela; and PS3 and PS4—restricted to Colombia and Venezuela respectively [18]. These authors did not include any isolates from the Brazilian Midwest. Another set of strains were investigated in parallel by other groups, and it was observed that the Pb01 strain did not cluster in any *P. brasiliensis* phylogenetic species reported at that time [19]. The isolation and microbiological characterization of the *Paracoccidioides* Pb01 strain were carried out at the Instituto de Patologia Tropical e Saúde Pública, Federal University of Goiás (IPTSP-UFG) by Dr. Maria do Rosário at the beginning of the 1990s. Since this strain was collected in Goiás and presented a unique intron insertion at the hsp70 gene [20], researchers screened the genetic background of 54 strains from Midwestern Brazil, aiming to find the shared genomic patterns [21]. Seventeen isolates from Mato Grosso, Goiás and a single occurrence in Ecuador were found to carry the same genomic insertion compared to the Pb01 strain, and these were named “Pb01-like” [21]. It was found that the Pb01-like strains were phylogenetically distinct from the *P. brasiliensis* species complex using PSC. These authors also demonstrated that conidial cells from strains belonging to the “Pb01-like” group were morphologically longer than the barrel-shaped *P. brasiliensis* conidia. This key finding led to the proposal of a new species comprising the Pb01-like population and this was named *Paracoccidioides lutzii* as an honor to the disease’s discoverer, Adolpho Lutz [22]. These observations were vital to understanding and explaining the serological immunodiagnostic challenges in PCM patients from Midwestern Brazil infected by *P. lutzii*, which sometimes was mis-diagnosed as tuberculosis. In the same fashion, the three other phylogenetic species within *P. brasiliensis* strictu sensu were proposed as follows: *P. americana* (PS2), *P. restrepiensis* (PS3) and *P. venezuelensis* (PS4) [23].

Through an initiative of the Broad Institute and several researchers, the reference genome of three species of *Paracoccidioides* were sequenced using Sanger Technology: Pb18 (*P. brasiliensis*), Pb03 (*P. americana*) and Pb01 (*P. lutzii*). The structural genomes ranged in size from 29.1 Mb to 32.9 Mb and encoded 7610 to 8130 genes. The comparative genomics of members of the *Onygenales* order contributed to understanding the clinical, biological and genetic differences among the species of *Paracoccidioides*. They also allowed phylogenetic studies to be carried out that estimated the divergence of this genus from other dimorphic fungi. This work provided, furthermore, a collection of unique genes and metabolic pathways conserved among *Paracoccidioides* dimorphic relatives [24]. More recently, the genome of 77 strains representing the five species of *Paracoccidioides* was sequenced using short-read Illumina technology and used for evolutionary analysis. The results reinforced that all *Paracoccidioides* species are reciprocally monophyletic, and gene flow (or hybridization) between species is infrequent [25,26]. Today, five structural draft genomes of *Paracoccidioides* isolates (Pb18, Pb03, Pb01, PbCnh and Pb300) have been sequenced, assembled, annotated and deposited in genomic databases. To improve and standardize the current genomic functional annotation, the *Paracoccidioides* genomic database (ParaDB, http://*Paracoccidioides*.com/) was recently launched, in which all data were compiled and manually curated, and can now be accessed for any *in silico* analysis. The availability of this resource will undoubtedly facilitate post-genomic studies of the *Paracoccidioides* [27,28].

From the 1990s, molecular studies aiming to explore the biology of *Paracoccidioides* had begun, even before the deduction of the full genomes. In 1995, Goldani et al. [29] cloned and sequenced the first genomic fragment related to a surface glycoprotein from *P. brasiliensis* for diagnostic purposes and differentiation of *P. brasiliensis* clinical isolates. In the following year, the gene encoding the 43 kDa glycoprotein (gp43) was characterized and cloned [30]. Historically it is important to note that, this immunodominant antigen of *P. brasiliensis* found in patients with PCM, was first described by Yarzabal L et al., as band E [31]. Later, this band was elegantly characterized by Dr. Travassos’ group, as the gp43 antigen. In the following decade, a significant advance in the understanding of *Paracoccidioides* sp. biology took place. Many studies have explored at the proteomic and genomic levels the differences between the two forms of the fungus, the infective (mycelial and saprophytic) and the pathogenic (yeast), as well as the morphogenetic events that culminate in the temperature-dependent dimorphic transition. Salem–Izaac et al. [32] identified more homogeneous protein synthesis patterns and gene expression in yeast cells than in the mycelial phase after evaluating several *P. brasiliensis* isolates. Through two-dimensional protein electrophoresis, Cunha et al. [33] identified and characterized differentially expressed proteins from each dimorphic stage of the fungus (Pb01); among them, PbM46, an enolase-like protein, was very abundant in the mycelium while PbY20 was only found in the yeast phase, which later was identified as a member of the flavodoxin-like WrbA family [34]. In parallel, Venâncio et al. [35] identified about 20 differentially expressed genes between *P. brasiliensis* yeast and mycelium cells and three regulated cDNAs during the transition from the infective to the pathogenic phase by using the differential display (DD) approach.

By *in silico* electronic subtraction, the expressed sequence tag (EST) sequencing project was started in 2001. Researchers characterized the functional genome of the *Paracoccidioides* Pb01 isolate, actually known as *P. lutzii*. The project resulted in the sequencing of 6022 genes differentially expressed between the forms of mycelium and yeast, and boosted the understanding of the metabolic and molecular events that Pb01 induces in its lifecycle, which enable it to adapt to the conditions found in the natural environment as a saprophytic form or in the host in its pathogenic phase. This study also provided categorized and comprehensive data related to cell cycle, stress responses machinery, signal transduction pathways, virulence genes, and drug targets for this pathogen [36,37,38,39].

At the same time, by EST sequencing, researchers from southeastern Brazil (São Paulo state) started the transcriptomic characterization of isolate Pb18, which is a *P. brasiliensis* stricto sensu individual. By sequencing 4692 expressed genes and comparative analysis with the *Candida albicans* database, these authors pinpointed potential homologues of *P. brasiliensis* essential for virulence and pathogenicity [40]. Among preferentially expressed yeast genes, some were required for metabolism, signal transduction pathways, growth and morphogenesis, and sulfur metabolism [41].

The abundant data generated by the functional genome projects of *P. brasiliensis* and *P. lutzii* (at that time it had not yet been defined as the *P. lutzii* species) were pivotal for further studies exploring the molecular mechanisms developed during the temperature-dependent dimorphic transition of these fungi, which is considered the main morphogenetic event required for the establishment of the disease [42,43]. Aiming to take the next step in understanding the metabolic adaptation of *P. lutzii* within the host, Tavares et al. [44] evaluated the gene expression of yeast cells after six hours of interaction with peritoneal murine macrophages. From 1152 genes evaluated by cDNA microarray technology, 152 genes were differentially transcribed in this condition. The collected data show that the fungus (Pb01) has a strong capacity to adapt to the harsh phagocyte environment, modulating the genes required for glucose and amino acid assimilation/production, cell wall remodeling, and oxidative stress response to evade and counteract the immune system. This plasticity guarantees its survival and continuation in the host.

Bailão et al. [45] identified by cDNA Representational Difference Analysis (cDNA-RDA) several genes required by yeast cells for dissemination via the hematogenic route. Among these, genes related to copper and iron metabolism were found, in addition to those necessary for cell wall/membrane remodeling. Next, Bailão et al. [46], when exposing Pb01 yeasts cells to blood and plasma, also observed changes of the transcriptional profile response to these conditions that would mimic the host environment. Other studies prompted a greater understanding of the survival and adaptation of *Paracoccidioides* in different sites and environments encountered inside the host [47,48].

Amid the progress made by transcriptomes, the genetic manipulation of *Paracoccidioides* was still something to be mastered. Two studies were published, the first in 2004. Here, Leal et al. [49] describe the technique of genetic transformation, mediated by *Agrobacterium tumefaciens*, to insert into the *P. brasiliensis* genome the gene responsible for Hygromycin B resistance. In parallel, Soares et al. [50] reported electroporation to deliver the Hygromycin B resistance gene to the Pb01 genome. Both studies reported low transformation efficiency and low mitotic stability of the recovered transformants. Among the factors that can be attributed are the multi-budding and multinuclearity of *Paracoccidioides* yeast cells observed among the isolates used as background strains.

It was only in 2009 that the first functional study of genes was published, to the enthusiasm of the *Paracoccidioides* scientific community. Almeida et al. [51] used antisense technology to knock-out the expression of CDC42 in *P. brasiliensis* yeast cells, an important protein for the control of yeast cell growth and virulence. The authors reported a 49%–88% efficiency in silencing the CDC42 gene. Despite this breakthrough, there is still no efficient gene deletion system based on classical genetic techniques and homologous recombination. The scientific community looks forward to seeing the CRISPR/CAS9 system, which is widely used for functional study of genes in fungal kingdom members, being applied to *Paracoccidioides* shortly. Even with this delay in the development of appropriate molecular tools compared to other human pathogenic fungi, several studies have been published in the past few years, using the gene expression modulation approach described by Almeida et al. [51,52,53,54,55,56,57,58,59,60].

**Figure 1:**
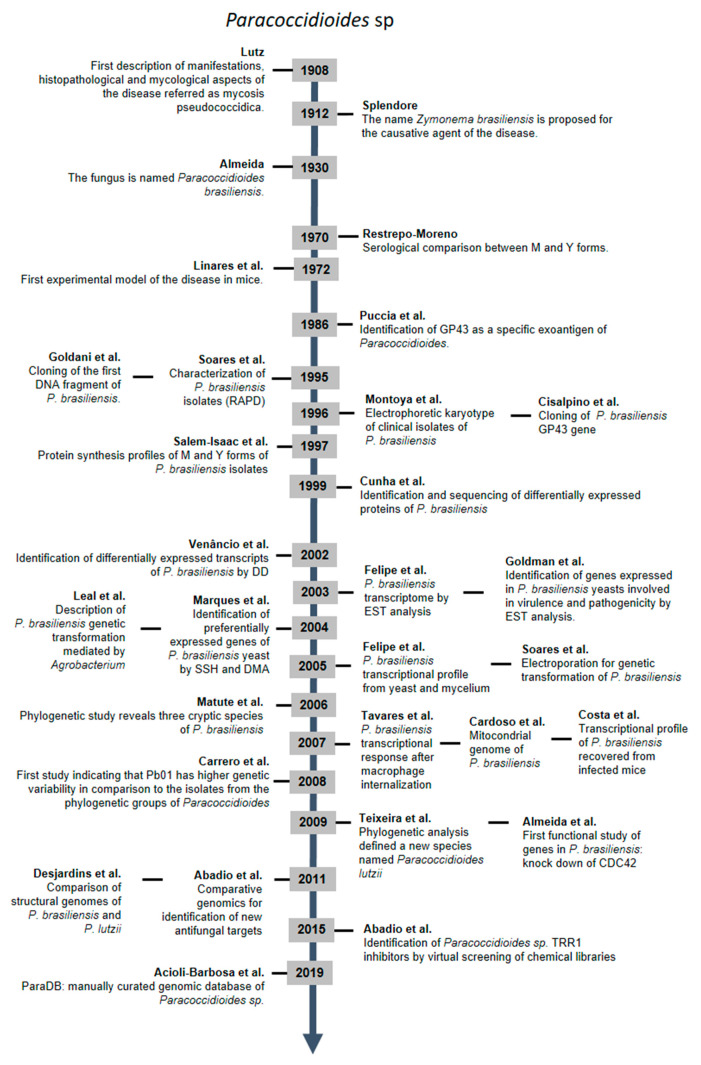
Primary studies related to *Paracoccidioides* sp. from the first description of the disease to the present day. The timeline herein illustrated for *Paracoccidioides* findings was based on a Pubmed database using the term “*Paracoccidioides*” as a query.

From the first description of the disease in 1908 by Lutz to the present day, a period of more than one century, much progress has been made in understanding the life cycle of this human pathogen. The development of molecular techniques boosted the number of publications related to *Paracoccidioides* and PCM. Today, more than 2300 published studies can be explored on the Pubmed database (https://pubmed.ncbi.nlm.nih.gov/), which clearly demonstrates the enormous efforts and impressive advance of researchers in understanding this pathogen and its disease. Figure 1 highlights the pioneering studies that paved the way on this long journey. There is still much to clarify and to explore. In this article, we will discuss how all these data accumulated to date can contribute even more to the development of new antifungal strategies to expand the therapeutic range for fighting the disease.

## 2. Search for New Therapeutic Options—A Journey

### 2.1. A Starting Point—Available Therapeutic Options

The achievements of the primary research on *Paracoccidioides* are undeniable, a history built by various hands. However, issues related to the treatment of patients with PCM still have a distance to travel. PCM treatment is restricted to three groups: polyenes, sulfa derivatives and azoles [61]. Generally, treatment consists of two phases: induction and maintenance. The induction treatment corresponds to the immediate control of the signs and symptoms and the fungal burden reduction to recover cellular immunity, a fundamental step for therapeutic success. The drugs used in this phase are usually more active and of intravenous administration but have more adverse side-effects. Therapy maintenance is usually performed with oral administration drugs and more extended treatment regimes. The reduction of antibody titers is generally a criterion used to start this phase. The treatment usually continues until cure criteria are reached, seeking to reduce the risk of disease recurrence. These criteria are based on four parameters: clinical (absence or regression of disease signs and symptoms), mycological (negative direct mycological examination), radiological (stabilization of pulmonary radiological image patterns), and immunological (antibody titer—negative or stabilized at low levels) [5].

Amphotericin B (AmB), a drug of the polyene class, has a fungicidal effect. The mechanism of action of Amphotericin B, the most commonly studied and well-known, is the interaction with ergosterol, the main sterol in the plasma membrane of fungi. In this case, there is the formation of pores and, consequently, the leakage of small ions and water molecules [62]. Despite studies using computerized modeling of the formed channel [63], other AmB action models have been proposed [64,65]. Anderson et al. have suggested that amphotericin B activity is due to the ergosterol depletion from the lipidic bilayer of the yeast membrane by large extra-membranous aggregates of AmB [64]. Besides, Mesa-Arango et al. [65], demonstrated that reactive oxygen species are essential mediators in the fungicidal effect of AMB. Since 1958, AmB has been a recommended drug for severe PCM cases. In recent years, it has been considered the antifungal of choice for the treatment of PCM, especially in the attack phase. However, it should be noted that this drug has several limitations, such as administration only by intravenous route and nephrotoxicity [66,67]. Although there are currently liposomal forms of Amphotericin B (LAmB), these present high costs, and maintain several adverse effects, such as fever, chills, tachycardia, tachypnea, and hypertension or hypotension [68]. However, there is no doubt that LAmB has a significantly improved toxicity profile compared with conventional AmB. This fact has encouraged several clinical studies with LAmB, in different situations in which treatment with AmB is clinically indicated, but the conventional formulation cannot be administered [69].

Several sulfa derivatives have been used since 1940. Sulfamethoxazole-trimethoprim (SMX-TMT) or cotrimoxazole is frequently used, especially in the maintenance phase [70]. This drug is distributed by the Brazilian public healthcare system and its use offers advantages such as low cost, acceptable toxicity profile, the achievement of adequate concentrations in several organs and availability by both oral and intravenous routes [5]. However, therapeutic failure has been described in 5% of patients, and the main disadvantage is the need for long-term treatments (more than 12 months), followed by adverse effects such as hypersensitivity reactions, leukopenia, megaloblastic anemia and thrombocytopenia [70,71].

In the same fashion, a randomized study performed in three Brazilian hospitals showed a similar therapeutic effect between itraconazole, ketoconazole and sulfadiazine [72]. The antifungal activity of triazoles is predominantly by CYP51 inhibition, a cytochrome P450 enzyme (sterol 14-alpha-demethylase), which prevents the conversion of lanosterol to ergosterol [73,74]. The therapeutic success of itraconazole in several patients with active PCM was reported, with a significant reduction in inflammatory infiltrates. The role of itraconazole in the pulmonary fibrosis control caused by PCM is still unclear, since few reports in the literature are available. Among them is one in which fibrosis became more evident after therapy, reaching 81% of patients treated [75]. In addition, the use of itraconazole involves several drug interactions: Warfarin, Calcium channel blockers, Carbamazepine, Cyclosporine, Simvastatin, Midazolam, Rifampicin, Sirolimus, Tracolimus and several antiretroviral drugs [5]. It is important to highlight that co-infection reports of PCM and tuberculosis are described in the literature, as well as of HIV patients with PCM. Thus, drug interaction is a limiting factor that should be taken seriously. Despite the advent of newer-generation triazoles, such as voriconazole and posaconazole, itraconazole is considered the main azole derivative used in clinical practice, with satisfactory results in non-hospitalized PCM patients [76,77]. This generation of antifungals can be considered as potential substitutes for itraconazole; however, they are still high cost and new clinical evidence needs to be published [5,66]. Another point to consider is that several azoles have renal excretion. In this sense, the dose should be adjusted in patients with renal disease [5].

The cell wall is in fact a drug target with great therapeutic potential. Glucan synthase (fks1) is listed among the potential targets for the further development of antifungals [78]. A recent review has reaffirmed the potential of this target [79]. However, the cell wall of *Paracoccidioides* is altered when this pathogen changes from mycelium to yeast form. β-1,3-glucan is replaced by almost entirely α-1,3-Glucan when this species enters the pathogenic yeast phase [80]. Thus, echinocandins showed no promising antifungal activity in the in vitro assays against *Paracoccidioides* sp., especially against yeast [81,82]. In addition, the most recent inhibitors Ibrexafungerp [83] and rezafungin [84] have not yet been evaluated for in vitro or in vivo activity in the PCM model.

In the specific case of PCM, even though treatment achieves success using the available therapeutic options, limitations related to nephrotoxicity, prolonged treatment and inability to control pulmonary sequelae are a reality among patients. Fortunately, the technological progress of the last few decades has made a large number of genomes from human fungal pathogens available. Therefore, the advances that the “-omics” era provided are undeniable, and a start has been made in which basic knowledge is widely used in applied research, such as new antifungal development [78,85,86,87,88,89].

### 2.2. Comparative Genomics Searching for Drug Targets

The development of antifungal agents with a broad action spectrum and minimal human toxicity is an urgent demand [87]. Currently, several approaches are available in the search and development of drugs [90,91,92,93]. Planning based on structure and mechanism of action has proven to be an efficient and less expensive strategy for developing new drugs [94]. A critical study showed that the most common drug targets are enzymes, particularly oxidoreductases, transferases and lyases. These protein targets are involved in binding, signaling and cellular communication. Usually, they are found in the cell membrane and in the cytoplasm, and are rarely present in organelles [95].

**Figure 2:**
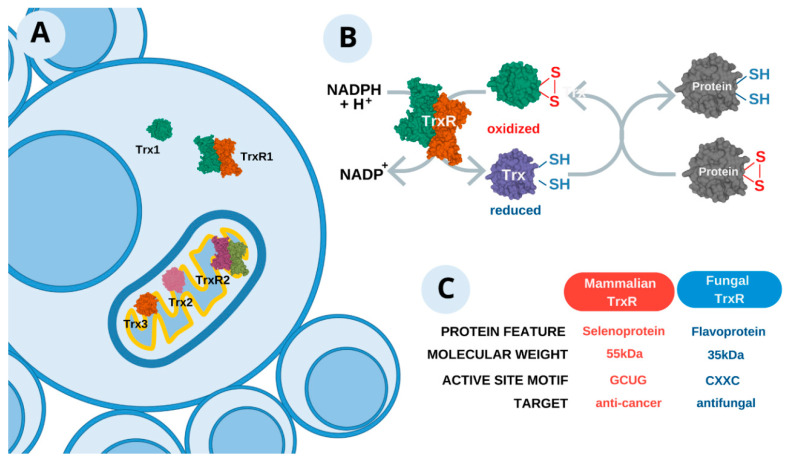
Thioredoxin system, an important means to control oxidative stress. (**A**) Cyto-localization of the thioredoxin system in *Paracoccidioides* sp. Three isoforms of thioredoxin were identified, namely Trx1 (cytoplasmatic) and Trx2 and Trx3 (mitochondrial). Thioredoxin reductase (TrxR) also participates in this system, which presents two forms, one in each compartment. (**B**) Schematic representation of the mechanism of action. The oxi-reduction reaction occurs so that cellular proteins are constantly available to control situations of the reactive species inside the fungus. (**C**) Comparison from thioredoxin systems between human and fungi.

Comparative genomics is an important tool for the initial step in searching for new compounds for treating severe mycotic diseases, including PCM. One strategy for searching for new antifungal targets is identifying essential genes that are important for the cell viability of the pathogen. The access to the complete genomic sequences of pathogenic fungi, including *P. brasiliensis* and *P. lutzii*, allowed target genes to be identified using bioinformatics techniques [78]. A comparative genomic analysis was performed using 57 orthologous genes present in *P. brasiliensis*, *P. lutzii*, *C. albicans*, *Aspergillus fumigatus*, *Blastomyces dermatitidis*, *Coccidioides immitis*, *Cryptococcus neoformans* and *Histoplasma capsulatum*. Among these selected genes, 55 are essential for *C. albicans* and/or *A. fumigatus* [96,97] and two are required for fungal survival within the host [98,99]. The alignments of these gene sequences and manual curation following the criteria, such as essential or needed for fungal survival, found in all the pathogenic fungi, and absent in humans, provided a list of 10 potential antifungal drug targets [78]. This work proposed a starting point in the search for new therapeutic options to treat fungal infection, cited more than 63 times, according to the metrics of Web of Science. Since that time, essential genes, pathogen-specific metabolic pathway enzymes and virulence and pathogenicity factors have been widely explored [87,100,101]. Thus, identifying essential genes preserved in several pathogens, which has also been used as a strategy, pointed to a very accurate way forward when selecting proteins from the thioredoxin system as potential drug targets (Figure 2A).

### 2.3. Thioredoxin System—A Promising Approach

#### 2.3.1. General Definition

Prokaryotic and eukaryotic cells have different redox systems, and among them is the thioredoxin system [102]. In this complex system, two elements are essential: the protein thioredoxin (Trx) and the enzyme thioredoxin reductase (TrxR, EC 1.6.4.5, named in many articles as Trr1). Trx are thermostable and low molecular mass proteins. The structure of this protein has a fold consisting of a central B-sheet, with five flanked by four α-helices. The Trx is an antioxidant known as a redox-sensitive molecule with a highly conserved active site (-Cys-Gly-Pro-Cys-), and catalyzes oxidation-reduction reactions by electron transfer dynamics (Figure 2B) [103]. This protein is reduced by TrxR, a selenocysteine containing protein and belonging to the flavoprotein class which binds flavin adenine dinucleotide (FAD) and nicotinamide adenine dinucleotide phosphate (NADPH) [103,104,105,106,107]. The pattern of binding and electron transference between NADPH and its FAD cofactor, besides the presence of TrxR (which catalyzes the reduction of Trx), are important for the maintenance of the reduced form of Trx [106], since most of the actions of Trx are performed when it is in the reduced state, stable and functional [108]. The active site of TrxR is found in the binding domain to NADPH, and the transfer of electrons from NADPH to the FAD-binding domain is essential for protein stability [109]. When the FAD domain receives electrons, it promotes protein rotation to allow the electrons to transfer to the active site and, in this way, the transitory binding of the NADPH-mediated TrxR to the oxidized Trx occurs [110]. The reduced Trx acts as a protein disulfide reductase and, when the active dithiol site interacts with oxidized cysteines of proteins, there is a thiol-disulfide exchange process forming an oxidized Trx [106].

The molecular weight of TrxRs classifies them into two important groups (Figure 2C). Those of high molecular weight (55 kDa for each subunit) are present in higher eukaryotes, and contain a selenocysteine in their active site [107]; furthermore, they can reduce various substrates in addition to thioredoxin [106]. This protein is part of the class I family of the pyridine nucleotide-disulfide oxidoreductase and has an active redox site composed of a seleno-protein forming the Gly-Cys-Sec-Gly domain. This is required to form a selenony-sulfide in the oxidized enzyme which is reduced by the active site of the other subunit in the dimeric enzyme [111]. In comparison, those of low molecular weight (35 kDa for each subunit) are present in prokaryotes, archaea and lower eukaryotes, such as fungi [106]. This enzyme is a member of the class II family of the pyridine nucleotide-disulfide oxidoreductase [112]. In the redox system, they are restricted to the reduction of thioredoxin [107] and, in addition, do not have selenium in the active site, leaving only cysteine [113]. Despite the similar function of isoforms, the molecular structure is different and these characteristics are essential for target selectivity [114].

The functions of the thioredoxin system are directly involved with cellular homeostasis. Various pieces of scientific evidence demonstrate the importance of this system in redox cell balance, repair of mutagenic DNA, cell growth and survival [106,115,116]. In mammals, three isoforms of Trx and TrxR have already been identified, differentiated by cyto-location. Trx1 and TrxR1 are present in the cytoplasm and are dominant forms [117,118]. In the mitochondrial location, Trx2 and TrxR2 are present [117,118]. Another Trx isoform localized in the mitochondria is Trx3. This 14-kDa protein was described in *Saccharomyces cerevisiae* containing the characteristic thioredoxin active site (WCGPC) [119]. Trx3 possesses two additional cysteine residues Cys57 and Cys6, overlaying the activity of the glutathione reductase system [120]. The sequences of all Trx and TrxR isoforms were predicted for *Paracoccidioides* sp. (Figure 2A).

In humans, increased Trx expression has already been seen in different types of cancer such as colorectal [121], lung [122], pancreatic [123], and gastric [124]. Increased oxidative stress increased in cancer cells may contribute to reduced apoptosis, up-regulation of cell growth, increased invasion, or even regulation of angiogenesis. Therefore, a strategy used in anti-cancer therapy is to obtain inhibitors that work selectively on the cytosolic isoform of thioredoxin reductase, reducing damage in normal cells [125].

#### 2.3.2. Fungus Selectivity

Research using the thioredoxin system as a target for treatment for fungal infections has increased in recent years [97,114,126,127,128,129,130,131]. The identification of essential genes is a strategy that elucidates new targets for antifungal therapy, and when widely conserved in fungi, there is an increased interest in acting as new targets for drug design [97]. Blocking the thioredoxin system is an excellent strategy that makes the fungus vulnerable to reactive oxygen species [129] and, therefore, its performance is essential for the vitality of fungi such as *C. neoformans* [126], *C. albicans* [114] and *A. fumigatus* [97].

Studies with *C. neoformans* have confirmed that this protein is induced during oxidative stress and showed that Trr1 is an essential gene for this species. *C. neoformans* TrxR is present in mitochondria and cytoplasm, participating imperatively in virulence [126,132]. The knowledge of the three-dimensional crystallized structure of this protein has been established, and it is a key point for future studies aimed at understanding the structural properties, catalytic activity and molecular mechanisms of Trx in *C. neoformans* [133]. As for *C. neoformans*, studies also show the importance of essential genes for the fungal genus *Aspergillus*. Hu et al. [97] identified the trr1 gene as essential for *A. fumigatus* vitality, and in addition this characteristic directly reflects the virulence of the pathogen [131]. For *A. fumigatus*, the thioredoxin system also acts in the maintenance of cellular redox homeostasis and, therefore, TrxR also presents a potential target for new antifungals [134]. Although other authors have already mentioned TrxR as a good target for antifungal therapy against *C. albicans*, it was only in 2016 that Godoy et al. [127] demonstrated the structural and functional characterization of the recombinant thioredoxin reductase from *C. albicans*. This recombinant protein effectively induced anti-CaTrxR antibodies that reduced the fungal burden in a disseminated candidiasis experimental model.

Historically, the Trx system is an important drug target for *Paracoccidioides* spp. [78,114,128,129,135]. The thioredoxin system investigation started in 2003 [35] in which genes that were supposedly involved in oxidative stress response expressed in *P. brasiliensis* were identified, including the trx gene. Additionally, Abadio et al. [78] used the comparative genomic tool and outlined the identification of potential antifungal therapy targets. Four drug targets were selected, including the enzyme Trr1. Recently, Oliveira et al. [129] showed the presence of Trr1 in the *P. lutzii* cell wall, highlighting it as an important target for therapeutic antibodies. The accumulated evidence that the TrxR enzyme of the thioredoxin system is required for the intracellular redox balance and, consequently, influences fungal viability, prompted researchers to search intensely for specific inhibitors against TrxR as a strategy for the development of new antifungals.

### 2.4. In Silico Strategy for New Antifungal Development

**Figure 3:**
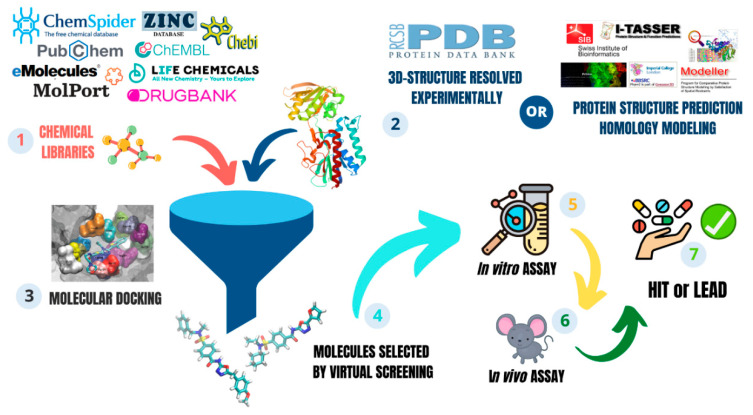
Drug design by *in silico* approaches. This process can be divided into at least seven steps. (1) Prepare the molecules libraries with filters such as Lipinski’s rule. Currently different databases are commercially available: https://www.molport.com/shop/index; http://www.chemspider.com/; https://zinc.docking.org/; https://www.ebi.ac.uk/chebi/; https://reaxys.emolecules.com/index.php; https://go.drugbank.com/; https://www.ebi.ac.uk/chembl/; https://pubchem.ncbi.nlm.nih.gov/; https://lifechemicals.com; (2) Prepare the target protein, either from the experimentally solved protein database (https://www.rcsb.org/) or by homology modeling (https://salilab.org/modeller/; https://zhanglab.ccmb.med.umich.edu/I-TASSER/; http://www.expasy.org/resources/swiss-model; https://robetta.bakerlab.org/; https://www.schrodinger.com/prime; (3) Analyze the *in silico* data in search of new molecules (4) Perform the experiments in vitro; (5) Analyze the results in vitro to start point (6) in vivo experiments; (7) Compounds that meet all the requirements can sequence with the hit-to-lead process.

Computational methods have come to play a significant role in drug design. Simulations based on the target structure bring a greater chance of success for drug development in less time [136] (Figure 3). The target prediction for drug discovery can be based on structural models available in databases, resulting from X-ray crystallography or NMR spectroscopy [137]. The best binding sites are identified by computational tools, according to physical-chemical properties, and thus the docking for selection of inhibitors can start by virtual screening [138]. The number of three-dimensional structures that have been resolved experimentally is growing exponentially, totaling 171,588 structures deposited by November 2020. This situation is not the same for fungi, much less for the genus *Paracoccidioides*. The Protein Database statistical data (https://www.rcsb.org/stats) indicate that fungal proteins represent about 6% (10,295 structures). Until now, only one structure from *Paracoccidioides* has been solved by X-ray crystallography, a nucleotidyl-transferase-like antigen, named Pb27 [139].

Thus, by homology modeling, protein structure prediction is a reality for the groups working with *Paracoccidioides* spp. Indeed, Abadio et al. [77] obtained three-dimensional structures of two proteins by homology modeling from *P. lutzii*: thioredoxin reductase and alfa-1,2-mannosyltransferase. The three-dimensional models allowed the identification of relevant information about these proteins, such as the conserved domains and the residues of the active site [77]. The 3D-structure of other *Paracoccidioides* proteins was built by *in silico* tools for virtual screening application, such as homoserine dehydrogenase (EC 1.1.1.3) [140], chorismate synthase (EC 4.2.3.5) [141] and isocitrate lyase [142].

#### 2.4.1. Virtual Screening

Virtual screening allows a search for several chemical libraries of small molecules and selects those that best interact with the specific target’s catalytic site [94,143,144,145]. The application of this technique is well accepted, mainly in the lead discovery phase [146,147]. Unlike the empirical choice of compounds, the selection of these specific target compounds reduces the amount of in vitro and in vivo assays performed to prove their activity, reducing costs, as well as the time spent comparing with chosen compounds without a rational selection [148,149]. The search for potential inhibitors of target enzymes can be performed in different chemical libraries, with thousands of molecules. After selection, hit compounds could then be synthesized and acquired for in vitro and in vivo validation (Figure 3). In recent years, the identification of thioredoxin system inhibitors by virtual screening has been a success story.

In collaboration with Maigret’s group (Loria, France), the best twelve small molecules that interact with the Trr1 from *P. lutzii* were virtually selected. These molecules were purchased and three presented specific antifungal activity against *P. lutzii* and *P. brasiliensis*. By using enzymatic assays, the recombinant thioredoxin reductase from *P. lutzii* was also inhibited by these molecules [114]. Afterward, molecules with an extended action spectrum for other fungal species of worldwide relevance—*Candida* and *Cryptococcus*—were selected as potential ligands of the active conformation of Trr1 by virtual screening. Crystallography studies revealed that the NADPH and FAD domains are located on opposite sides of the molecule, requiring a significant conformational change for electron transport to occur [150,151]. In this approach, two small molecules (from the oxadiazoles class) were selected as Trr1 inhibitors of three pathogenic fungi: *Candida* spp., *Paracoccidioides* spp. and *Cryptococcus* spp. [152]. These new compounds proved to be broad-spectrum with antifungal activity against *C. albicans*. The inhibitory activity in vitro was 64.9 μM, without cellular toxicity and, most importantly, significantly reduced the fungal burden on the kidneys of mice in the candidemia experimental model [128]. One of these oxadiazole compounds, named LMM11, showed activity against *C. krusei* with MICs ranging from 65 to 129.9 μM, and it also succeeded in reducing colony-forming unit (CFU) recovered from the kidneys of infected mice with this clinically-relevant pathogen, mostly involved in hospital infections [130]. The two oxadiazoles (LMM5 and LMM11) selected against TRR1 showed a range of inhibitory action from 2.03 to 71.99 μM against nine isolates of *Paracoccidioides* spp. They also showed a significant reduction in CFU recovered from infected and treated mice and found no toxicity in a murine PCM model [135].

Another molecule selected by virtual screening against *Paracoccidioides* protein was CP1, a derivative of quinolinic alkaloids. This molecule targeting chorismate synthase from *P. brasiliensis* showed excellent antifungal activity against many clinical isolates from *Paracoccidioides* spp. [139]. In this same sense, Bagatin et al. [140] selected inhibitors of homoserine dehydrogenase (HSD) from a library of natural products, which showed specific antifungal activity against *Paracoccidioides* spp. The two new amino acid derivative compounds, HS1 and HS2, exhibited MICs of 260.9 and 123.4 μM, respectively, against *P. brasiliensis* (Pb18 isolates) and 246.81 μM against *P. lutzii*. In addition, synergic activity with itraconazole was observed [140]. Given the promising results using HSD as a target, a new virtual screening was performed to search for optimized compounds. The molecule HS9 (Zinc2123137) presented fungicidal activity that was more promising than HS2, with MIC values of 23.44 μM and low cytotoxicity in human cell lines [153]. Two recent studies indicate the synthesis of lead molecules from these hit molecules selected by virtual screening [154,155].

**Figure 4:**
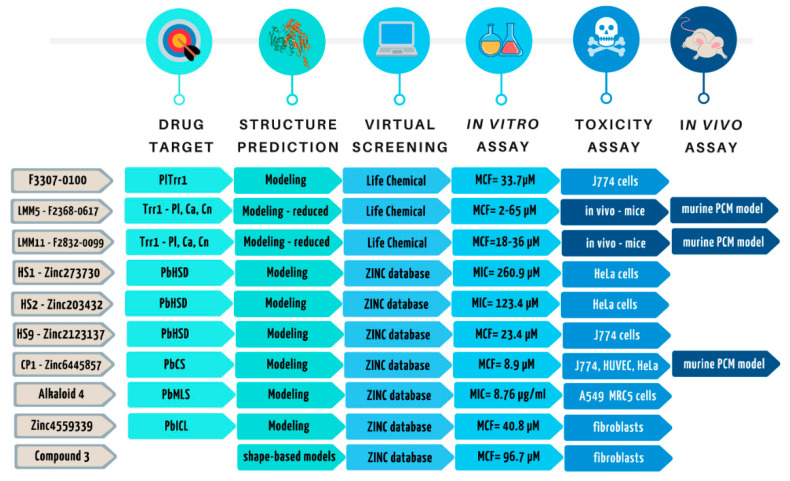
Comparison of selected molecules by virtual screening. Ten different molecules showed promising values of antifungal activity and absent of toxicity in vitro. Only three molecules were tested in an experimental model of paracoccidioidomycosis (PCM). F3307-0100 [114], LMM5 and LMM11 [135], HS1 and HS2 [153], HS9 [140], CP1 [141], alkaloid 4 [146] Zinc4559339 [142], Compound 3 [158].

From the theoretical model of malate synthase, four compounds were selected by virtual screening against this enzyme present in fungi and absent in mammals. The compounds selected presented moderate antifungal activity, with MIC values varying from 8.76–249.57 μg/mL. Unfortunately, these compounds present specific toxicity in cell lineage [146]. A promising hit molecule was selected by virtual screening against alpha-1,2-mannosyl transferase from *P. lutzii* [156]. This molecule, named MOL3, presented in vivo antifungal activity against *Paracoccidioides* spp. (data not shown), and for many *Candida* species, especially *Candida parapsilosis* [157]. Virtual screening to search for compounds chemically related to a molecule with known antifungal activity may be an interesting approach. Three chalcone derivatives, selected by this approach, showed promising results in vitro, with MIC values varying from 2.9–12.6 μM against different *Paracoccidioides* spp. isolates and low cytotoxicity [158]. Recently, isocitrate lyase from *P. brasiliensis* was modeled by homology modeling, and the virtual screening was performed with many natural compounds from the ZINC database. The best-selected compound (ZINC4559339) presented values MIC of 19.1 μM in *P. brasiliensis* with a fungicidal effect at this concentration [142]. Therefore, ten different molecules showed promising values of antifungal activity and absent of toxicity in vitro (Figure 4).

#### 2.4.2. Drug Repositioning

Drug repurposing or repositioning aims at a new indication for drugs already approved to treat other diseases and/or whose mechanism of action or targets are already known [159]. This methodology directly reduces time and costs for the drug to reach the clinical trial stage [160], since, for example, data on pharmacokinetics and pharmacodynamics may already be available [161]. Satisfactory results have already been shown in therapy for parasitic diseases [162] and cancer [163]. Drug repositioning appears as a viable alternative for discovering new drugs against mycoses [164] and other neglected diseases [165].

Concerning new antifungals in the literature, we found a significant number of studies on targeted drug repositioning in mycoses caused by *Candida* spp. [166] or *Cryptococcus* spp. [167]. However, with dimorphic fungi, such as *Paracoccidioides* spp., there are still few studies described. In this context, the antiviral raltegravir showed activity against *Paracoccidioides* isolates [168]. Ligand-based and structure-based (molecular docking) computational methods were used for repositioning. The selected compounds were chemically similar to two-hit compounds previously selected against thioredoxin reductase, with promising antifungal activity. All the six compounds evaluated showed antifungal activity against the genus *Paracoccidioides*, particularly raltegravir (MIC: 36 μM for *P. brasiliensis* and MIC: 72 μM for *P. lutzii*). Additionally, this antiviral drug showed promising antifungal activity against experimental murine paracoccidioidomycosis, with a significant reduction of the fungal burden and decreased alterations in the lung structure of treated mice [168].

Oliveira et al. [169] developed computational drug repurposing—chemo-genomics—to identify anti-PCM activity drugs. The goal of chemo-genomics is to establish the molecular relationships of ligands and targets using genome data [170], within the concept that “similar targets have similar ligands” [171]. Studies with chemo-genomics *in silico* have already appeared satisfactory for drug repositioning in species of parasitic *Schistosoma mansoni* [172]. The old-drugs that have been repositioned belong to antineoplastic (vistusertib and BGT-226) and azole compounds (bifonazole, luliconazole, butoconazole and sertaconazole). These compounds presented significant antifungal activity [169].

### 2.5. Drug Targets Discovered from Natural Products

Natural compounds and derivatives are still widely used in the search for molecules with antimicrobial potential [101]. The variety of bioactive secondary metabolites present in plants has been an inexhaustible source of compounds with antifungal activity [173], including against PCM. Since 1993, the antifungal activity of ajoene (natural garlic compound) against *P. brasiliensis* has been known [174]. In 1997, the same authors suggested that the anti-*P. brasiliensis* activity may be due to the blocking of phosphatidylcholine biosynthesis [175]. Maluf et al. [176] confirmed the activity in vivo of ajoene in experimental PCM and Thomaz et al. [177] indicated the additive effect between ajoene and sulfamethoxazole/trimethoprim.

Recently, OMICs tools have helped describe the action mechanisms of these natural products and their possible molecular targets. Oenothein B (OenB), a macrocyclic hydrolyzable tannin dimer extract of *Eugenia uniflora* L. (a Brazilian Cerrado plant), inhibited the 1,3-β-D-glucan synthase (*Pb*FKS1) transcript accumulation in the *P. brasiliensis* isolate Pb01. This natural compound interferes with the fungal cell morphology [178]. Other enzymes that also participate in cell wall synthesis have been modulated by OenB, such as those expressed by GLN1 and KRE6 genes [179]. Enzymes that participate in cell wall synthesis and those involved in the protein mannosylation process have been described as targets of drugs by Abadio et al. [78].

A series of β-carboline alkaloids isolated from *Rubiaceae* and *Apocynaceae* species showed activity against *Paracoccidioides* spp. and *in silico* experiments suggest interaction with malate synthase (MLS) [180]. PbMLS acts on the glyoxylate cycle and in the allantoin degradation pathway [181] and it is considered a multifunctional and versatile protein [182]. Argentilactone is the principal constituent of the essential oil from the Brazilian savanna plant *Hyptis ovalifolia*. This compound and its semi-synthetic derivatives were able to inhibit the activity of isocitrate lyase from *P. lutzii* (PbICL), yeast cell growth, and differentiation from mycelium to yeast [183]. This is another enzyme of the glyoxylate cycle that could be exploited as a drug target [184].

Antifungal activity of thiosemi-carbazide camphene derivative (TSC-C) in isolates of *Paracoccidioides* spp. was also demonstrated. The transcriptome analysis suggests this derivative was related to the induction of the ROS formation in *Paracoccidioides* spp. [185]. By chemo-proteomics, Borba et al. [186] suggest that thiosemi-carbazides (TSCs) and TSC-C were multitarget compounds with an effect on several essential mechanisms for *Paracoccidioides* such as electron-transport chain, cell cycle, ROS formation, cellular metabolisms and mycelium-yeast transition. Thus, the crucial enzymes and metabolic processes were described as potential targets of promising antifungal molecules, mainly natural compounds and synthetic derivatives.

## 3. Nanotechnology as an Approach for Alternative Therapies

Different strategies to improve PCM therapy are under development, many using the benefits promoted by nanotechnology (Table 1), whether carrying molecules that act as immunomodulatory boosts such as the peptide P10 [187,188], a DNA vaccine [189] or the scFv fraction from a recombinant antibody [190]. These carriers may also carry conventional drugs, such as amphotericin B [191,192] and itraconazole [191]. Such strategies were able to improve murine treatment for PCM significantly, demonstrating that the formulation of these compounds in nanostructured systems had benefits such as reduced dosage and side effects as well as positive modulation of the immune response of animals in the fight against fungal infection.

Nanostructured drug delivery systems have been developed considering the principles of nanotechnology, which deals with materials or tools on a small scale (10^−9^ m). In this dimension, materials exhibit different physical and chemical properties, allowing an efficient association with other materials, including antifungals [194,195]. For the construction of these systems, a diversity of materials is used, chosen from the characteristics required for the final formulation.

Because of the small size of these drug delivery systems, the molecules (drugs and natural compounds) associated with these nano-formulations also perform differently, reaching body regions that would not be possible or would be reached less efficiently if administered in a conventional formulation. These nanostructured systems are appreciated for being used to deliver molecules that can be easily degraded when administered in vivo, such as peptides or unstable molecules [196]. In the case of polymeric nanoparticles, these molecules can be surrounded by a polymeric matrix protecting them from the action of enzymes present in the physiological environment, thus increasing their half-life [197]. In addition, nanoparticles can be functionalized by binding, on their surface, those molecules that exhibit tropism for specific organs or cells, resulting in a sophisticated and efficient drug delivery system.

As far as we know, one of the first studies using nanotechnology for drug delivery in experimental PCM was carried out by our group [191]. In this strategy, it was possible to incorporate the antifungal amphotericin B in a polymeric formulation prepared using the copolymer of lactic-co-glycolic acids (PLGA) and functionalized with dimercapto-succinic acid (DMSA), which presents tropism to the lungs [198]. Amphotericin B was chosen because it is a drug that, despite its excellent fungicidal activity, in the conventional formulation of sodium deoxycholate causes unwanted and severe side effects, such as nephrotoxicity and hepatotoxicity [199].

In this drug delivery system, it was possible to incorporate three times the daily dose prescribed for the treatment of PCM using amphotericin B [191]. This strategy proved to be effective and it was possible to administer the treatment every three days since the degradation of the PLGA was slow, releasing the drug for up to six days. Complementary studies to evaluate the biodistribution of nanoparticles labeled with technetium-99 showed that these nanoparticles were distributed to different organs, not only in the lungs, as expected by the fact they were functionalized with DMSA [200]. These nanoparticles may have slowly degraded in organs and the amphotericin B was released in the circulation, exerting the antifungal activity.

The association of amphotericin B with magnetite nanoparticles pre-coated with a double layer of lauric acid was also investigated [192]. This system proved to be an alternative to the conventional formulation of sodium deoxycholate for this drug, since in vivo treatment results were similar. An interesting point for this type of nanostructure is its ability to reach extremely specific regions of the organism. In some situations, such as granulomatous lesions, magnetic nanoparticles can be submitted to an alternating magnetic field for better drug action at the site of infection [201]. This technology is still under development and improvement, but it can bring benefits when generating heat, since an alternating magnetic field influences these magnetic nanoparticles. However, the toxic potential of this type of nanostructure must be considered, mainly concerning the coating and functionalization of this type of nanoparticle [202], as they are prepared from iron oxide and, considering the long duration of the antifungal treatment with multiple doses, can cause accumulation and be harmful to the patient.

Polymeric nanoparticles functionalized with DMSA were also evaluated to encapsulate itraconazole [193]. The nanoencapsulation of fungistatic compounds, such as itraconazole, is an important therapeutic option because of the nature of these drugs, whose mechanism of action works by inhibiting the growth of new cells, which could be considered a risk for the development of drug resistance [203,204]. The incorporation of itraconazole in these nanoparticles promoted a more significant accumulation in the lungs, liver and spleen, sites of *Paracoccidioides* spp. infection, which increases the efficiency of this class of antifungals.

Some materials used in the preparation of nanoparticles slightly stimulate the immune system. Once in contact with biological fluids, the nanoparticles may be captured by macrophages [205,206]. In some cases, this is desirable, when working with vaccines or immunomodulatory molecules. Some studies have explored the potential of nanoparticles as an adjuvant in the treatment of PCM.

In one of these studies [190], the adjuvant capacity of PLGA was investigated for single-chain variable fragments (scFv) of a recombinant antibody for gp43, the main antigen for PCM [207]. When incorporated within PLGA, scFv presented an increase in its protective capacity in animals challenged with *P. brasiliensis*. The results were also observed when the P10 peptide, also a fragment of gp43, was incorporated into PLGA [187], in which a 20-fold reduction in the amount of P10 needed to trigger the immune response was achieved. It is important to highlight that when complexed within the polymer, other adjuvants such as Freud’s were not needed, although these are necessary when using the free molecules. This is auspicious, since there is no need to use adjuvants in a possible therapy using this strategy.

Whereas both scFv and the P10 are very unstable molecules for application in a physiological environment, as they can be easily degraded by peptidases, once incorporated in these polymeric nanoparticles they are protected, while remaining in circulation for a prolonged time [196]. On the other hand, nanoparticles may be phagocytized and thus deliver the antigen directly to phagocytes, allowing efficiency in triggering the protective immune response [205]. This type of nano-formulation can be administered by different routes, such as intramuscular or intranasal. In a study comparing the immunomodulatory potential of a DNA vaccine, DNAHsp65, for the treatment of PCM [189], liposomes and PLGA nanoparticles were evaluated for delivery of DNA Hsp65. Both formulations were shown to be effective in treating infection, although they received a dose four times lower. The systems proved to be efficient, but the group treated with liposomes received it intranasally, making this vehicle more attractive for antifungal therapy in humans.

The intranasal administration route was also chosen to evaluate a nanostructured formulation for the P10 peptide considering the mucosa present in the nasal route. Thus, polymeric nanoparticles were prepared with chitosan [188], Chitosan is a natural cationic polymer, biocompatible, biodegradable and mucoadhesive, which can interact electrostatically with the anionic mucin present in the airway [197]. The results indicated a 20-fold reduction in the amount of peptide needed to present an efficient protective immune response in animals. Considering the potential of nanotechnology presented by studies using nano-formulations for the treatment of PCM, it could be explored to develop an even safer and more efficient therapy for the treatment of diverse fungal infection.

## 4. Challenges and Prospects

Despite the relevance of fungal infections, especially with the increase in life expectancy of individuals affected by chronic degenerative diseases and, consequently, more people in conditions of immunosuppression, interest from the pharmaceutical industry is still limited. This fact is due to several issues, such as the absence of notification of fungal infections, the type of population affected and the endemic character of some mycoses, as is the case of PCM. Additionally, PCM was recognized as a neglected disease by the scientific community only a short time ago, not being included on the list of neglected diseases of the World Health Organization. Therefore, the development of new therapeutic options for fungal infections is a challenge for academia.

Historically, development of a new drug from laboratory research to its arrival on the market takes around 10 to 15 years and requires a high investment (Food and Drug Administration). The cost of a new drug is the sum of the values of the pre-clinical and clinical phase, and the cost of developing new drugs has increased over the years. Therefore, to place an antifungal drug in the big pharmaceuticals’ pipeline does not seem to be something that attracts interest. In this context, rational drug design has been pointed out as a cost-effective alternative to traditional approaches. The *in silico* methods have reduced the costs and time associated with the initial stages of drug development. These advances, which catalyze the drug design process, were only possible with the rapid development of computer hardware, software and algorithms. These tools allowed researchers to think about the possibility of making viable new therapeutic options for fungal diseases, among them paracoccidioidomycosis, the object of study of many researchers in Brazilian medical mycology.

This process was only possible with advances in basic research into this fungus. The contribution of researchers working with *Paracoccidioides* to progress in medical mycology is undeniable, as much data was generated in the areas of genomics, proteomics, *in silico* methodologies, nanotechnology, diagnostic tools and even vaccine development. Comparative genomics, as well as proteomics, enables studies and description of new drug targets that have been rapidly explored in the search for new therapeutic options. These approaches have contributed to a crucial point, which is the activity profile of molecules. Several authors have discussed the importance of antifungal activity as a fungicide. However, combining fungicidal activity and low side effects or toxicity is still a challenge when working with fungi, eukaryotic microorganisms that share very similar biological systems as hosts.

In fact, several publications and patents have described promising compounds and molecules for PCM treatment, some of which have already passed important stages, such as the initial pre-clinical trials. However, some bottlenecks must be overcome in order for these promising molecules to work more effectively and reach the next level, the much-dreamed-of clinical trials. First is the need for large quantities of these molecules, purchased or synthesized, to move forward in the next stages. Second is the need to develop pharmaceutical formulations that provide an adequate administration route and sufficient bioavailability. In addition, pharmacokinetic and pharmacodynamic studies, either through computer simulations or laboratory analysis, are essential. Finally, the costs need to be lower, so that all these obstacles can be effectively overcome.

To this end, researchers working specifically on paracoccidioidomycosis disease continue to search for new therapeutic options. Researchers continue to try to overcome the obstacles so that some of the studied molecules may be in the final stage of development in the near future and contribute to the patient’s well-being, which is the end of the chain. Research is focused on the individual, who needs appropriate treatment, capable of controlling the infection and the after-effects of PCM, with few or no side effects, in a fast and effective manner. A new therapeutic option against *Paracoccidioides* opens new perspectives for the treatment of other mycoses.

## Figures and Tables

**Table 1:** Different strategies using nanotechnology for the treatment of experimental PCM.

Type of Nanostructures	Encapsulated Compound	Reference
PLGA + DMSA	Amphotericin B	Amaral et al. [191]
PLGA + DMSA	P10 peptide	Amaral et al. [187]
Liposome and PLGA	DNA Hsp65 vaccine	Ribeiro et al. [189]
Magnetic nanoparticles	Amphotericin B	Saldanha et al. [192]
PLGA	scFv	Jannuzzi et al. [190]
PLGA + DMSA	Itraconazole	Cunha-Azevedo et al. [193]
Chitosan	P10 peptide	Rodrigues et al. [188]

PLGA: copolymer of lactic-co-glycolic acids; DMSA: dimercapto-succinic acid.

## Data Availability

No new data were created or analyzed in this study. Data sharing is not applicable to this article.
